# How effective are social distancing policies? Evidence on the fight against COVID-19

**DOI:** 10.1371/journal.pone.0257363

**Published:** 2021-09-22

**Authors:** Ulrich Glogowsky, Emanuel Hansen, Simeon Schächtele

**Affiliations:** 1 Department of Economics, Johannes Kepler University Linz, Linz, Austria; 2 Center for Macroeconomic Research, University of Cologne, Cologne, Germany; 3 Inter-American Development Bank, Washington, D.C., United States of America; East China Normal University, CHINA

## Abstract

To fight the spread of COVID-19, many countries implemented social distancing policies. This is the first paper that examines the effects of the German social distancing policies on behavior and the epidemic’s spread. Exploiting the staggered timing of COVID-19 outbreaks in extended event-study models, we find that the policies heavily reduced mobility and contagion. In comparison to a no-social-distancing benchmark, within three weeks, the policies avoided 84% of the potential COVID-19 cases (point estimate: 499.3K) and 66% of the potential fatalities (5.4K). The policies’ relative effects were smaller for individuals above 60 and in rural areas.

## 1 Introduction

Since its outbreak in Wuhan, the SARS-CoV-2 virus causing the respiratory disease COVID-19 has spread across the globe [1–3]. To prevent human-to-human transmission of the virus, many governments have adopted social distancing (SD) policies. For example, more than 190 countries have implemented nationwide school closures [4]. These and similar policies aimed at reducing interpersonal contacts to dissipate the epidemic and, ultimately, save lives.

In this paper, we evaluate the effectiveness of the German social distancing policies in the fight against COVID-19. We offer two contributions. First, we provide a comprehensive analysis of the policies. We do not only estimate their impact on confirmed COVID-19 cases but also on fatalities. Additionally, we investigate if the policies affected certain socio-demographic groups more than others, and we use cell phone data to link the policies to changes in mobility behavior. Second, we propose a flexible quasi-experimental strategy that can be applied to many settings. At the core, it exploits variation in the spread of COVID-19 at the subnational level.

As for the policy variation, we focus on nationwide SD policies that the German federal and state governments jointly enacted in mid-March 2020. The most significant pieces of this policy response were Chancellor Merkel’s televised appeal for voluntary social distancing (March 12), the closure of schools, childcare facilities, and retail stores (March 16), and the implementation of a national contact ban (March 23). Our paper identifies the combined effect of all these policies. As the entire set of policies were simultaneously introduced in all German districts and within a short period of time, it is impossible to estimate their effects separately.

It is challenging to identify the effects of nationwide SD policies. The impact of such policies is the difference in an outcome of interest (e.g., confirmed cases) between states of the world with and without them. After the policy interventions, however, we cannot observe how the outcome would have developed without the policies. We tackle this problem with an extended event-study approach that exploits variation at the level of German districts (NUTS-3 regions; comparable to US counties). Some districts experienced a COVID-19 outbreak several weeks before the policies took effect; others were not yet affected. Hence, we can compare how the outcomes developed after a local outbreak without SD policies (former districts) and with SD policies (latter districts). This comparison identifies the policies’ effects if, in the counterfactual state without policy interventions, the outcome would have evolved similarly in both types of districts. We cannot test this assumption directly, but we verify its plausibility.

Three features render our approach and setting especially suited to estimate the policies’ effects. First, focusing on within-country variation lowers the potential for bias from heterogeneity in institutions, measurement, and populations. Particularly, in the first phase of the epidemic, all German districts faced the same policies and identical testing and reporting rules. Second, the German data are sufficiently granular for quantitative impact analysis: 401 districts with varying local outbreak dates report cases and fatalities to one federal agency. Third, the data quality is arguably high, and the expected share of undetected cases is lower than in most other countries [5]. The main reasons why epidemiologists expect a low share of undetected cases in Germany are low case fatality rates and extensive testing. As of April 22, Germany conducted 2.07 million tests (2.6% of the population).

**Fig 1:**
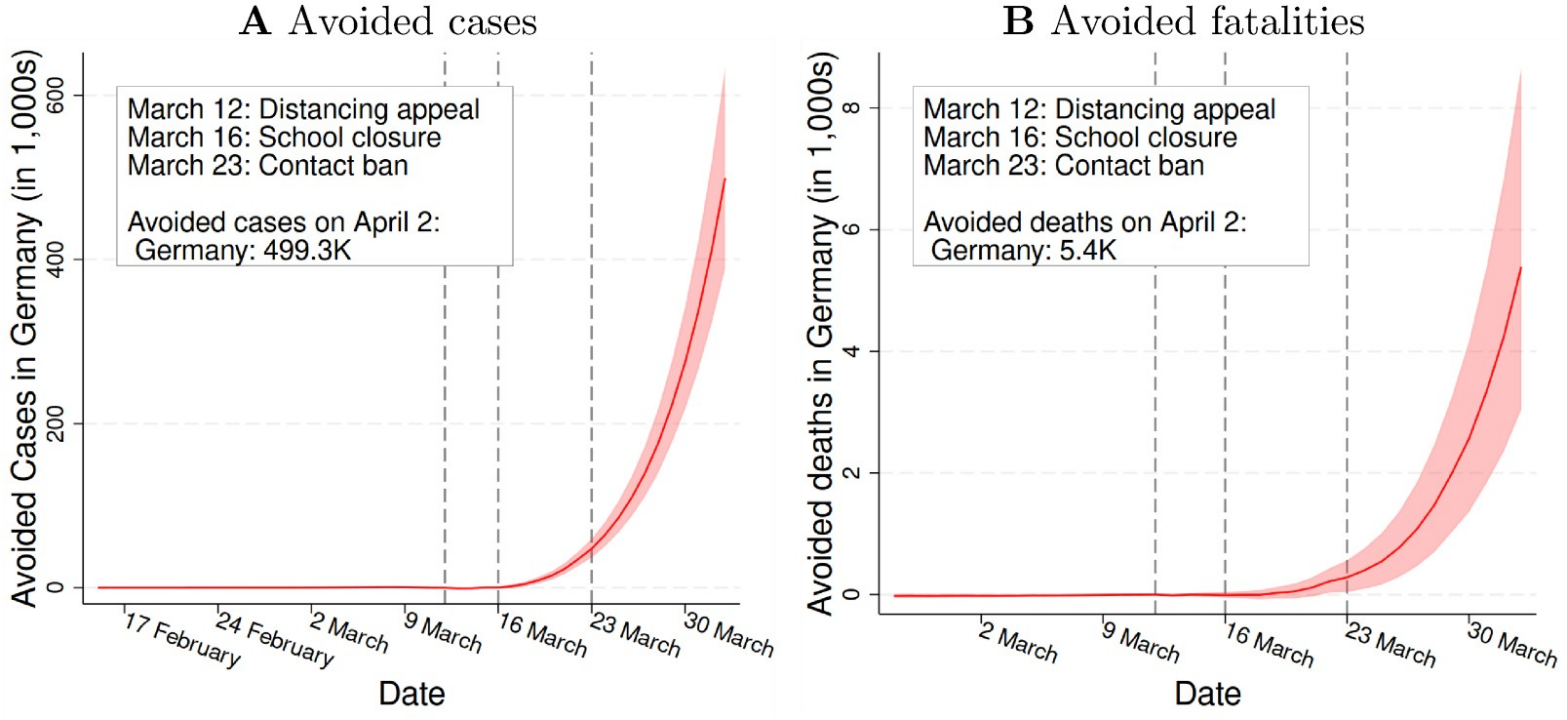
The effectiveness of social distancing policies in Germany. ***Notes***: Fig 1A and 1B summarize the main takeaway message of the paper. Fig 1A depicts our estimates on the number of cases avoided due to social distancing policies. Fig 1B depicts our estimates on the number of fatalities avoided due to these policies. The three vertical lines mark the Chancellor’s appeal for social distancing (March 12), the nationwide school closures (March 16), and the nationwide contact bans (March 23). The dashed lines represent 95% confidence intervals based on district-levelclustered standard errors.

Fig 1 gives a graphical account of our key results. It shows how the SD policies affected the number of cases (Fig 1A) and fatalities (Sub Fig 1B) within three weeks of implementation. According to our estimates, the policies avoided 499.3 thousand cases (95% CI: 389.4K-634.1K) and 5.4 thousand fatalities (95% CI: 3.0K-8.7K) until April 2. Put differently, the policies prevented around 84% of the confirmed cases and 66% of the fatalities that would have occurred without policy interventions by that time. The heterogeneity analysis shows that the policies’ relative effects were smaller for (a) the oldest age group (60+) and (b) in rural areas.

Furthermore, our analysis of cell phone data implies that, without SD policies, citizens would not have limited their social contacts to the same extent: According to our estimates, individuals reduced their mobility by about 30.7% with SD policies. Without them, they only would have lowered it by 3.9%. This suggests that the citizens limited their social contacts as intended by the German authorities.

By providing the first comprehensive evaluation of the German SD policies, we add to an ongoing public and scientific debate on whether SD policies contained the virus. In Germany, for example, many citizens consider the lockdown measures appropriate to fight the pandemic [6]. Other individuals, including German or US citizens, stage large-scale protests against them and question their effectiveness. The scientific debate on the policies’ effectiveness is not settled, either. Particularly, researchers have argued that the prevalent model-based evaluations of SD policies [7–15] suffer from methodological issues [16]. In particular, it has been argued that epidemiological models (a) are frequently weakly identified as they fit many parameters to a single time series [17, 18], (b) rely on too restrictive assumptions [19], and (c) often have limited predictive ability [20].

A recent suggestion to evaluate SD policies while avoiding these issues is to use quasi-experimental (instead of model-based) methods [21]. In this spirit, we propose a flexible and widely-applicable quasi-experimental approach that exploits district-level variation in the spread of COVID-19. We then apply this method to provide a broad analysis of SD policies, including their effects on individual behavior, confirmed cases, and fatalities. Hereby, we contribute to a small but growing literature that exploits quasi-experimental techniques to evaluate the effectiveness of non-pharmaceutical interventions [22–24]. Compared to our study, the corresponding papers have different focuses: They study fewer or other outcomes, employ different identification approaches, and investigate other policies. For example, one study investigates the effectiveness of travel restrictions [22], a second one studies the effect of SD policies on hospitalizations and cases [23], and a third paper examines the impacts of shelter-in-place orders on cases in the US [24]. Methodologically, these papers exploit policy variation across countries or regions in difference-in-difference models. Another study employs a similar empirical strategy to examine the role of social capital in the spread of COVID-19 [25]. In contrast to us, they do not focus on the impact of policies.

The paper’s structure is as follows: Section 2 describes the institutional background and Section 3 our estimation approach. Section 4 contains the results for mobility (Subsection 4.1), cases and deaths (Subsection 4.2), each with a description of the relevant data. The results section also features our heterogeneity analyses (Subsection 4.3) and robustness checks (Subsection 4.4). Section 5 concludes.

## 2 COVID-19 and social distancing in Germany

### 2.1 COVID-19 outbreak

In Germany, COVID-19 spread after the detection of two cases on February 25, 2020 (an earlier outbreak detected on January 27 had been completely contained). In the following weeks, the infection propagated to the entire country. On March 20, there were confirmed infections in all but one of the 401 German districts. S7 Fig in S1 File shows the distribution of district-specific outbreak dates. We define the local outbreak date as the first day when ten cases had occurred within two weeks. S8 and S9 Figs in S1 File consider other outbreak definitions.

### 2.2 Social distancing policies

**Fig 2:**
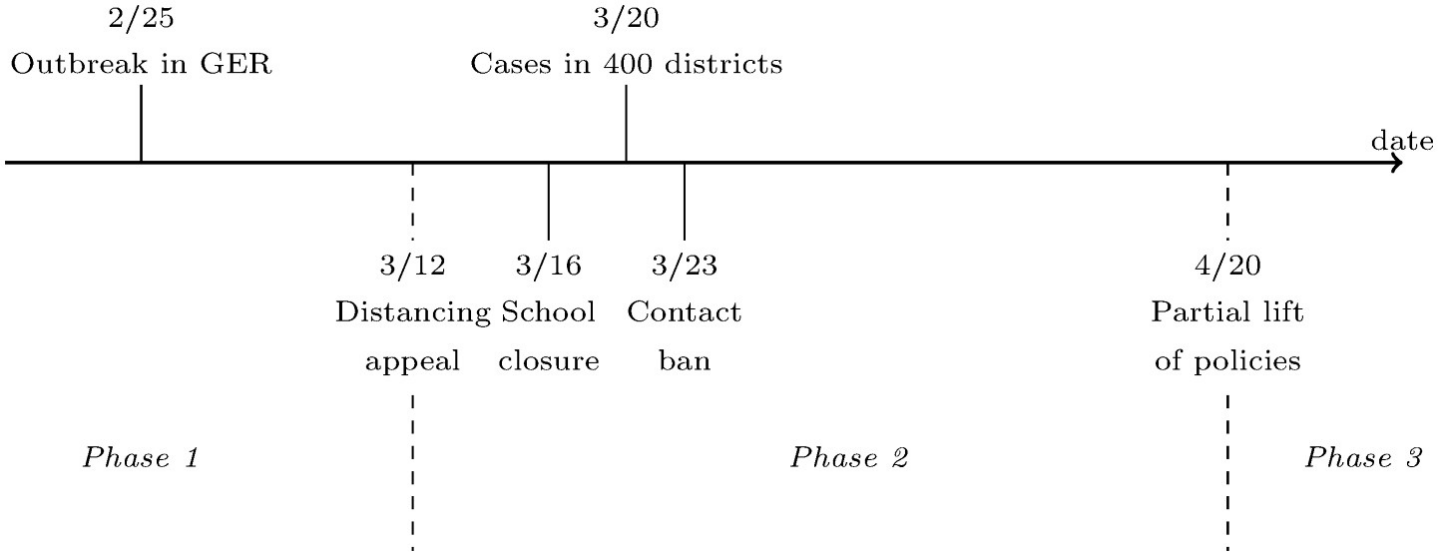
Timing of social distancing policies in Germany.

We classify the policy response during the first month of the epidemic into three phases (see Fig 2). In the first phase, starting with the detection of the first COVID-19 cases, the German authorities only took limited actions: They put infected persons under quarantine, recommended intensified hygiene practices to the public, and canceled large events with more than thousand participants around March 9.

In the second phase, the German federal and state governments agreed on additional simultaneous, nationwide containment policies to fight the epidemic. This phase began on March 12, when Chancellor Merkel appealed to all citizens to avoid social contacts whenever possible. Between March 13–15, the state governments announced the closure of schools, childcare facilities, and most retail stores starting on March 16. On March 22, they declared a strict contact ban: From March 23, meeting more than one person from outside the household was prohibited and keeping a minimum distance of 1.5 meters was required. As apparent, these policies’ goal was to limit the social contacts of German citizens. Henceforth, we refer to them as “social distancing policies.” Notably, our analysis identifies the combined effect of all these nationwide social distancing policies. In a different vein, data on internet search behavior suggests that citizens did not anticipate these interventions (see S16–S19 Figs in S1 File).

The third phase started on April 20, when the authorities gradually relaxed the policies.

### 2.3 COVID-19 testing

Official guidelines determine who qualifies for a COVID-19 test. During the study period, patients with flu-like symptoms were tested if they had been in contact with a person diagnosed with COVID-19 or in a high-risk area. This general rule applied in all federal states and remained almost unchanged during the sample period. After the virus had spread all over Europe, the authorities dropped the high-risk criterion on March 24.

## 3 Estimation method

Because it is impossible to observe the scenarios with and without SD policies simultaneously, one cannot estimate the policies’ effects by directly comparing outcomes between both states. Instead, we need to find a way to approximate the latter, counterfactual scenario. To that end, we present an extended event-study model [26–28]. While this section briefly introduces the event-study model, the S.1 Section in S1 File discuss in more detail how this model identifies the effects of SD policies.

### 3.1 Model

We estimate the following multivariate event-study regression with ordinary least squares (OLS),

$$\begin{array}{c} Y_{it}=\sum_{k\in \{\cdots ,-1,0,1,\cdots \}}\alpha_{k}\cdot 1\left[et_{it}=k\right]+\sum_{j\neq 3/11}\beta_{j}\cdot 1\left[t=j\right]+\varepsilon_{it}, \end{array}$$ (1)

where *Y*_*it*_ refers to the outcome of interest (e.g., confirmed cases). Moreover, *et*_*it*_ denotes the “epidemic time” in district *i* at date *t* (i.e., the number of days since the local outbreak of COVID-19). Regression (1) includes two sets of explanatory variables. The first set consists of binary (dummy) variables for each epidemic time before, at, and after the district-specific outbreak. Specifically, the dummy variable 1[*et*_*it*_ = *k*] takes the value one if the district-specific epidemic time *et*_*it*_ equals *k* and zero otherwise. The second set includes dummy variables for each day before and after the German SD policies started. The dummy variable 1[*t* = *j*] equals one if the calendar date *t* of the observation equals *j* and zero otherwise. We omit the dummy for March 11, the day before Merkel’s appeal. The last term in (1), *ε*_*it*_, represents the error term.

### 3.2 Interpretation of estimated parameters

While the *α* parameters account for how the outcome would have developed without the SD policies, the *β* coefficients capture the policies’ effects (henceforth, SD effects). To see why *β* captures the SD effects, note that the outcome with SD policies in district *i* at *t* is *Y*_*it*_. If the epidemic time in district *i* is *et*_*it*_ = *k*, the predicted outcome without SD is ${\hat{Y}}_{it}={\hat{\alpha }}_{k}$. Consequently, an estimate of the SD effect at *t* ≥ *March 12* is ${\hat{\beta }}_{t}=E_{i}[Y_{it}]-E_{i}[{\hat{Y}}_{it}]$. We can estimate $\hat{\alpha }$ and $\hat{\beta }$ separately because, conditional on the calendar date, there is variation in the districts’ epidemic times.

### 3.3 Further aspects

**Fig 3:**
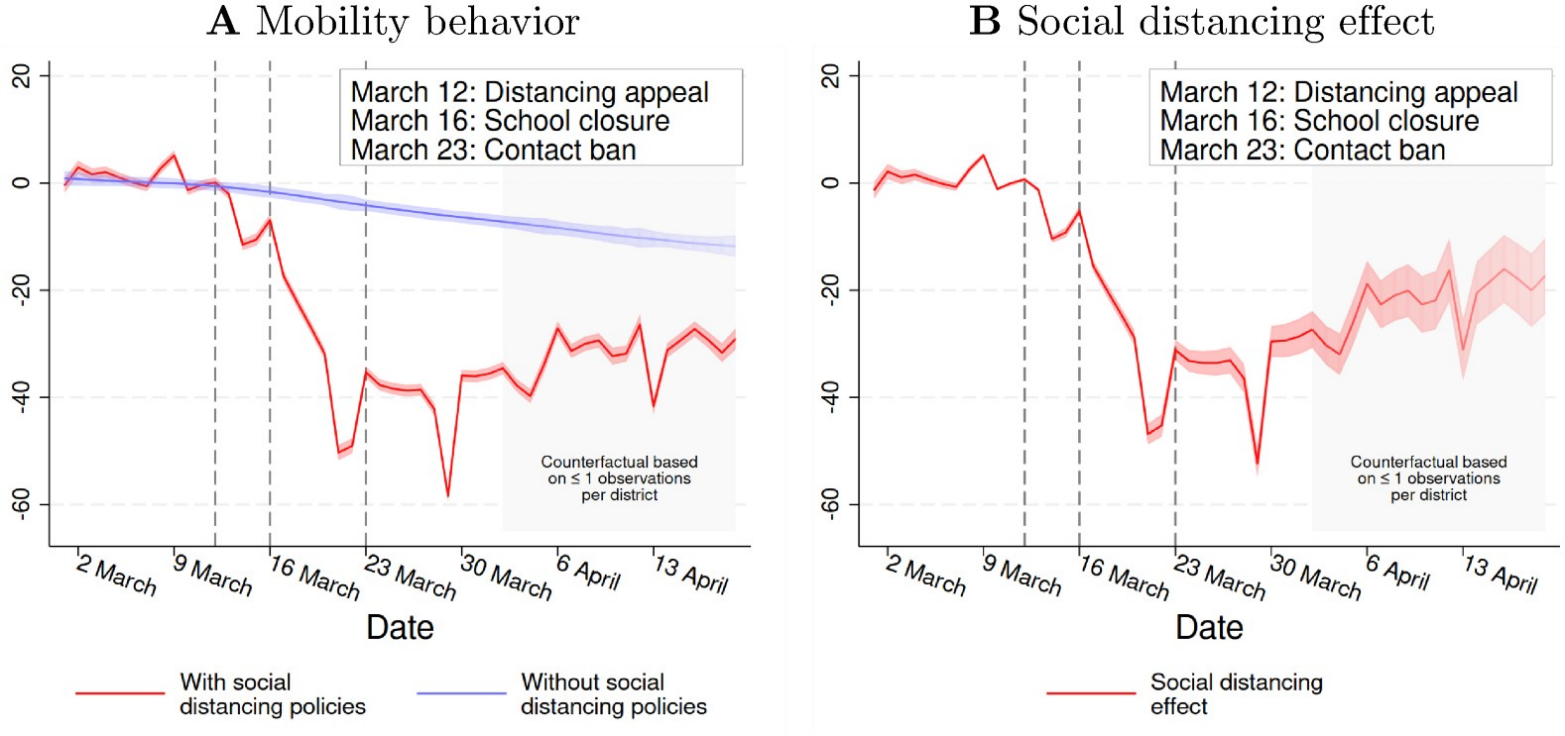
The social distancing effect on mobility. ***Notes***: Fig 3A and 3B show how social distancing policies affect mobility behavior. Fig 3A demonstrates how individuals’ behave in the realized world with social distancing policies (red line) and in the counterfactual world without SD policies (blue line). The spike on March 29 reflects very bad weather conditions at this particular day. Fig 3B shows the implied social distancing effect, measured in percentage points. The three vertical lines mark the Chancellor’s appeal for social distancing (March 12), the nationwide school closures (March 16), and the nationwide contact bans (March 23). The dashed lines represent 95% confidence intervals based on district-level-clustered standard errors.

Two aspects of our approach are essential to note. First, the identifying assumption (known as “parallel trends”) is that the expected outcome without SD policies would have been $E_{i}[{\hat{Y}}_{it}]$ for *t* ≥ *March 12*. Intuitively, this assumption implies that, without SD policies, the outcomes in districts with outbreaks after the intervention would have developed similarly as in districts with outbreaks before the intervention. We can conduct plausibility checks. Before the implementation of the policies, the estimated SD effects ${\hat{\beta }}_{t}$ should not differ from zero. Furthermore, prior to the intervention, the outcomes in districts with earlier and later outbreaks should be similar conditional on epidemic time. Both checks suggest that the assumptions hold (see Fig 3 and S2 Table in S1 File).

Second, the further away date *t* moves from the implementation date, March 12, the fewer no-SD observations we can use to estimate the counterfactual $E_{i}[{\hat{Y}}_{it}]$ and, in consequence, the treatment effect *β*_*t*_. To see this, note that, at the date of the policy intervention, the districts are at epidemic times between a lower threshold $\underline{k}$ and an upper threshold $\bar{k}$. Then, Δ days after the intervention, the districts have progressed to epidemic times between $\underline{k}+\Delta$ and $\bar{k}+\Delta$. Loosely speaking, we estimate the treatment effect by comparing the districts’ outcomes with SD to no-SD outcomes from before the intervention, but with the same epidemic time above $\underline{k}+\Delta$ (and below $\bar{k}$). Hence, the larger Δ, the smaller is the number of no-SD observations that we can use.

Until April 2, there is, on average, at least one no-SD control observation per district *i*. Therefore, our analysis focuses on the policy effects up to this date. For interested readers, we nevertheless provide estimates from an extended analysis until the policies’ relaxation on April 19 (see S1 File).

## 4 Results

### 4.1 Mobility

Did citizens limit their human-to-human interactions due to the SD policies? One approach to studying this question is to examine if the policies changed mobility [15]. The rationale is simple: Individuals who travel less or stay at home cut back the personal contacts outside their household.

#### 4.1.1 Measuring mobility

When cell phone users move, their phones switch cell towers to ensure connectivity. From these switches, providers can determine the number of trips starting or ending in a given geographic zone [29]. We obtained data on the number of trips at the district level from Teralytics, a business partner of Telefónica. Specifically, for each district *i*, *N*_*it*_ denotes the number of trips on date *t* in March or April 2020, and ${\bar{N}}_{i}\left(d\right)$ is the average number of trips on weekday *d* ∈ {Monday, …, Sunday} in March 2019. Our mobility measure on date *t* in district *i* is:

$$\begin{array}{c} \Delta N_{it}=(\frac{N_{it}}{{\bar{N}}_{i}\left(d_{t}\right)}-1)\cdot 100, \end{array}$$ (2)

where *d*_*t*_ is the weekday corresponding to date *t*. Hence, the measure adjusts for weekday-specific mobility patterns. Moreover, it has a simple interpretation: *ΔN*_*it*_ measures mobility relative to the number of movements in 2019.

#### 4.1.2 Results

To study the effects of the SD policies on mobility, we use measure (2) as the outcome of model (1). Fig 3 presents our results graphically. Fig 3A plots the estimated mobility behavior without policies (blue line) and the actually realized behavior with SD policies (red line). For each date *t*, the SD effect corresponds to the vertical difference between these two lines. Fig 3B shows this effect measured in percentage points. In both figures (and all following figures), the dashed lines represent 95% confidence intervals based on district-level clustered standard errors.

Three observations stand out. First, before the start of the second phase on March 12, individuals hardly changed their behavior relative to the baseline year 2019 (see red line in Fig 3A). This suggests that the cancellation of large events around March 9 did not affect mobility. Second, from March 12 on, citizens became less and less mobile. Shortly after Merkel’s appeal, they traveled slightly less. Mobility decreased more sharply and persistently, however, after the school and business closures on March 16. From March 16 to April 2, individuals traveled, on average, 30% less than in 2019. This reduction is six times larger than the estimated change without SD policies (-3.9%). Third, the effects of SD policies on mobility are large over the entire second phase, although they decrease over time (see Fig 3B). In sum, Fig 3 suggests that the SD policies reduced mobility considerably and, presumably, also social contacts.

#### 4.1.3 Further evidence

In the S1 File, we provide additional descriptive evidence that Germans became less mobile after the implementation of SD policies. For example, they reduced their trips to workplaces by more than 30% and used public transportation by about 50% less (see S14 Fig in S1 File).

### 4.2 Cases and fatalities

Next, we explore if SD policies effectively constrained the spread of COVID-19. Again, we focus on the period until April 2. S1 and S2 Figs in S1 File provide our estimates for the extended period until April 19.

#### 4.2.1 Measuring COVID-19 cases and fatalities

The district-level health offices are legally obliged to report confirmed COVID-19 cases and fatalities to the federal Robert Koch Institute, which collates and publishes these data daily [30, 31]. We use the data set provided on April 30, 2020. S5 and S6 Figs in S1 File show descriptive statistics. The data quality is comparatively high. First, the share of undetected cases is expected to be lower than in many other countries [5]. Second, all COVID-19 cases and fatalities are laboratory-confirmed. Third, all health offices apply the same testing and reporting criteria. Fourth, the data contain information on the day of the first symptoms for most cases and fatalities. For asymptomatic cases, the day of the first symptoms is set equal to the registration date. Additionally, we gathered state-level data on the numbers of conducted COVID-19 tests for robustness checks.

#### 4.2.2 Results for confirmed COVID-19 cases

**Fig 4:**
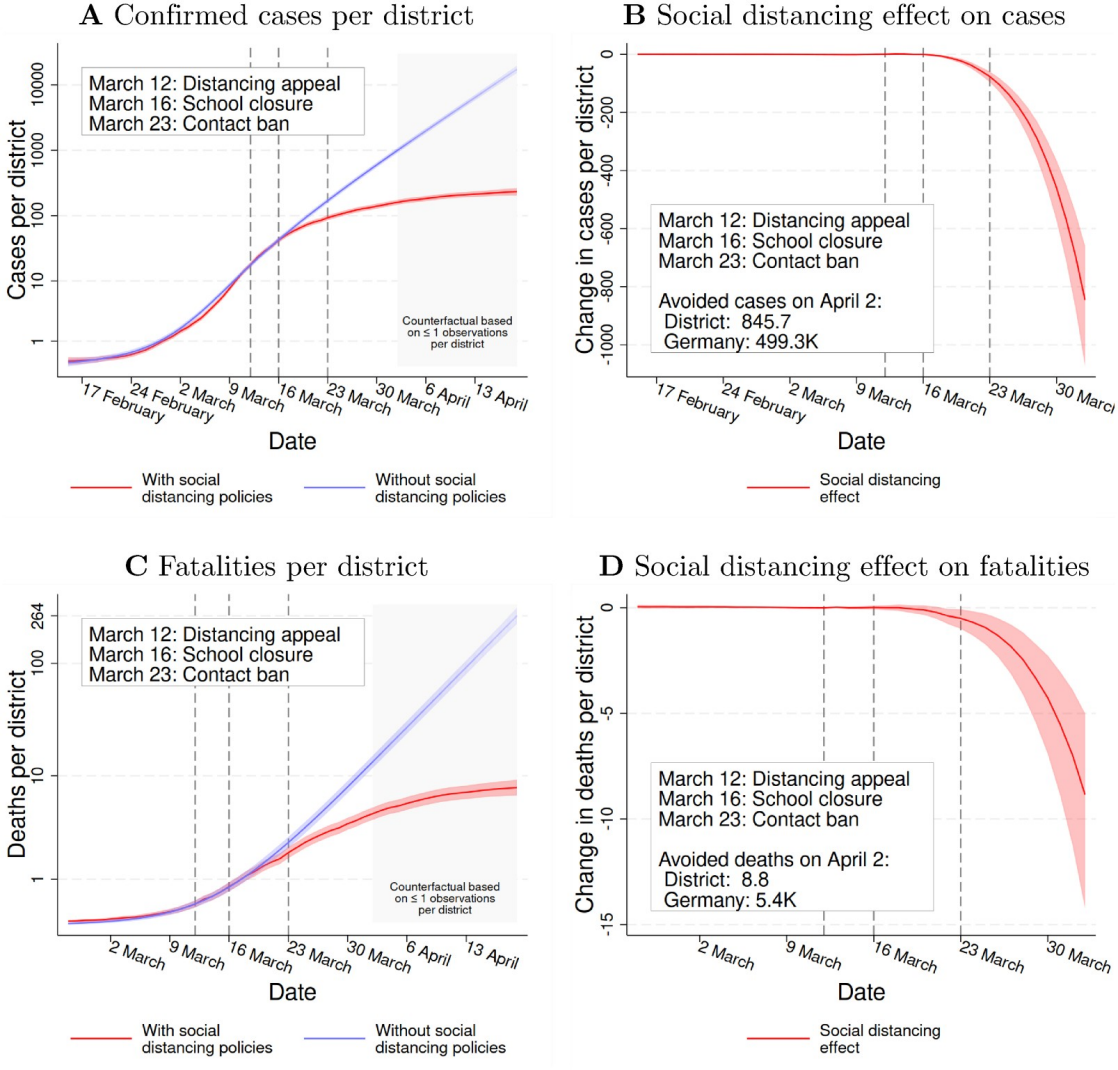
The social distancing effect on cases and fatalities. ***Notes***: Fig 4A and 4B show how social distancing policies affect confirmed COVID-19 cases; Fig 4C and 4D show how the policies affected fatalities related to COVID-19. More specifically, Fig 4A demonstrates the development of cases in the realized world with SD policies (red line) and in the counterfactual world without SD policies (blue line). It shows average cases per district, using a logarithmic scale. Fig 4B presents the implied social distancing effect on cases using a linear scale. Fig 4C presents the development of fatalities in the realized world with (red line) and in the counterfactual world without (blue line) SD policies. Fig 4D presents the social distancing effect on fatalities. The three vertical lines mark the Chancellor’s appeal for social distancing (March 12), the nationwide school closures (March 16), and the nationwide contact bans (March 23). The dashed lines represent 95% confidence intervals based on district-level-clustered standard errors.

To study the SD effects on COVID-19 cases, we use the inverse hyperbolic sine (IHS) of the cumulative number of confirmed cases in each district as the outcome in model (1). To simplify the interpretation, Fig 4 presents our estimation results re-transformed to cases (rather than IHS-values). Fig 4A displays how the confirmed cases per district truly evolved with SD policies (red line) and, according to our estimations, would have evolved without SD policies (blue line). We present the results on a log scale.

Importantly, our analysis is based on the day of the first symptoms. Therefore, if the blue line lies above the red one, our estimates imply that the number of individuals suffering from first symptoms at date *t* would have been higher without SD policies. Fig 4B depicts the corresponding effects of the SD policies on a linear scale. To make the timing of the policy effects more easily visible, the figure zooms in on the period until April 2. As the mean incubation period is 5–6 days [32], we do not expect to find significant policy effects before March 16.

The key insights from the Fig 4A and 4B are as follows: First, before the closure of schools, the case numbers with and without SD policies match closely (see Fig 4A). This finding suggests that our identifying assumption holds. Second, the growth rate of actual cases (red line) starts to diminish a few days after the start of the nationwide policy response, while counterfactual cases (blue line) continue to grow at a similar rate as before. Specifically, the first significant (yet small) SD effects appear on March 18, six days after Merkel’s appeal (see Fig 4B). Given the mean incubation period, the timing of the effects is hence in line with the policies’ implementation dates. Third, on April 2, our point estimate indicates that the SD policies reduced COVID-19 cases by about 84% or 846 cases per district. Converted to the national level, this estimate indicates that the SD policies prevented 499.3 thousand cases (95% CI: 389.4K-634.1K). The extended analysis suggests that the effects would have continued to grow strongly over time. Fourth, we can also interpret our results in terms of the reproduction number *R*, calculated according to the official methodology of the Robert Koch Institute [33]. After the policies’ introduction, *R* quickly decreased from above 2 to below 1 (see S12 Fig in S1 File). Our estimates suggest that, without SD policies, it would have stayed above 2 until April 2. In summary, the analysis implies that the SD policies effectively contained COVID-19.

#### 4.2.3 Results for fatalities

Next, we estimate model (1) with the IHS of the cumulative number of fatalities as our outcome. Recall that each fatality is reported together with the day of the patient’s disease onset. Hence, we study the effects of the SD policies on the number of (eventually) lethal infections that started on date *t*. Fig 4C and 4D present our estimation results for fatalities. The abscissa starts with February 26, the first day with ten or more eventually lethal infections.

The results are in line with those for confirmed cases: The fatalities in the SD and no-SD scenarios initially follow the same growth path (see blue and red lines in Fig 4C). A few days after the implementation of the policies, however, the scenarios diverge: While actual growth in fatalities slows down sharply (red line), the number of counterfactual fatalities continues to grow at a similar rate (blue line). Specifically, the SD effects are significant from March 21 on and increase strongly over time. We estimate that during the period March 11 to April 2, the SD policies decreased fatalities by 66% or 8.8 per district. Transformed to the national level, this estimate suggests that the policies reduced lethal cases with first symptoms until April 2 by 5.4 thousand (95% CI: 3.0K-8.7K). Again, the extended analysis hints at steadily growing effects over time.

### 4.3 Heterogeneity analyses

Some groups are at higher risk to suffer from severe COVID-19 progressions [32, 34]. International data show that hospitalization rates increase above 60 years of age. Furthermore, men seem to be at higher risk than women. Therefore, in the next step, we investigate how the SD effects differ (a) across age groups, (b) by gender, and (c) between urban and rural districts.

**Fig 5:**
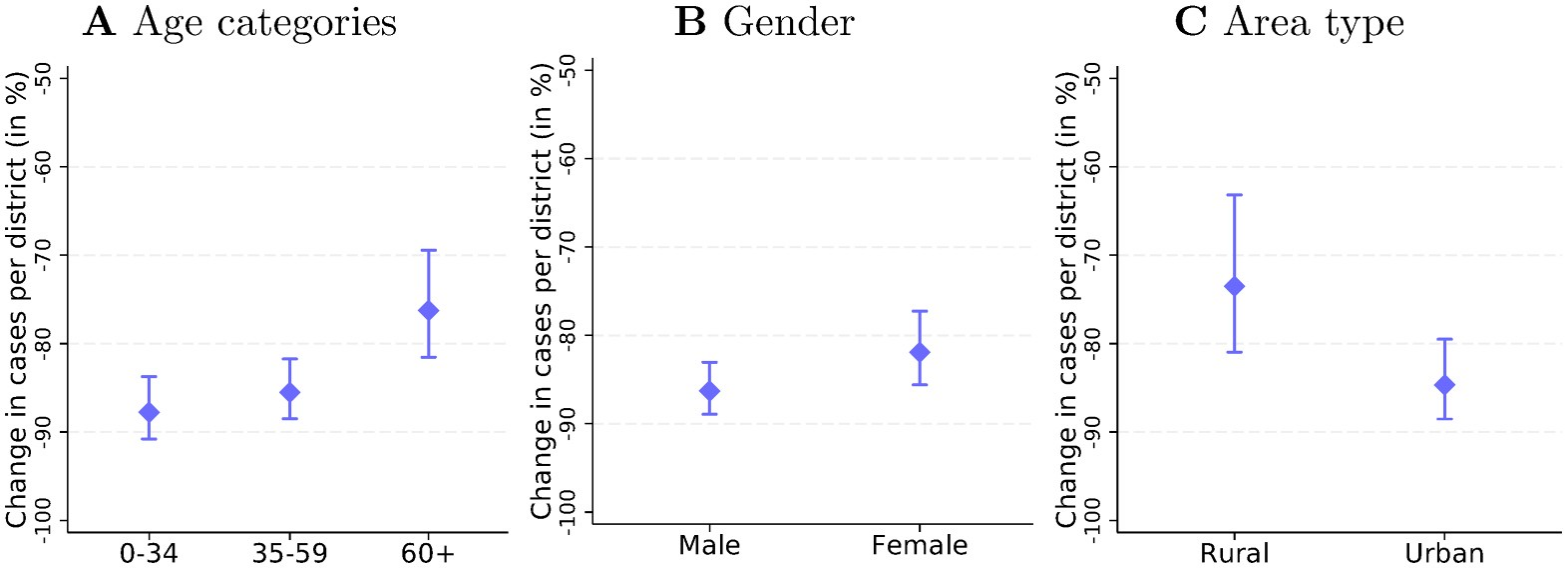
Heterogeneity of the social distancing effect on cases. ***Notes***: The three panels of Fig 5 show how the effect of the nationwide social distancing policies on April 2 differed across age groups (Fig 5A), by gender (Fig 5B), and between urban and rural districts (Fig 5C). The estimates rely on sample splits and show the percentage of confirmed COVID-19 cases avoided by the nationwide policies. The vertical lines represent 95% confidence intervals based on district-level-clustered standard errors.

Fig 5 shows the subgroup-specific SD effects on confirmed cases. The estimates rely on sample splits and indicate the percentages of cases avoided due to the policies until April 2. Three results emerge. First and foremost, we find large effects in all groups. The point estimates range from 76% to 88%. Second, Fig 5 presents evidence for age-group heterogeneity. The policies prevented 88% (182.5K) of the cases among individuals below 35 that would have otherwise occurred, 86% (257.5k) of the cases in the medium-age group, and 76% (76.9k) of the cases among persons of age 60 and above. The difference between the relative effects in the last and the two former groups is significant at the 5% level. The finding is in line with the observation that, after the policy intervention, the share of persons above 60 among all infected persons increased from about 20% (March 11) to 27% (April 2). This age heterogeneity seems plausible: Policies such as school and business closures likely have stronger implications for the working-age population and for children and their parents than for retirees. Third, we also find somewhat larger relative effects for urban districts than for rural districts and for men than for women. While the former difference is significant at the 10% level, the gender difference is not.

S3 Fig in S1 File shows the same pattern of subgroup-specific SD effects on fatalities. However, due to lower numbers, we cannot study some groups and the estimation uncertainty is higher. S4 Fig in S1 File extends the analysis until April 19. Again, the patterns are similar.

### 4.4 Robustness analyses

We probe the robustness of our results in various dimensions. First, we explore different definitions of a local outbreak. Second, we run additional regressions in which we control for the number of conducted COVID-19 tests per day. Third, we repeat our analysis using alternative outcome definitions. For example, we drop districts with zero cases or fatalities and estimate models in logarithms. We also apply the ln(1+ *x*) transformation to our outcomes. Fourth, we cluster the standard errors at the state level. S10 and S11 Figs in S1 File report the corresponding results. All conclusions remain substantially unchanged.

## 5 Conclusion

This paper provides evidence on the effects of the German social distancing (SD) policies on (a) individual behavior, (b) confirmed COVID-19 cases, and (c) fatalities. We show that, first, the SD policies affected individuals’ mobility. Second, we find that the policies sharply slowed down the spread of the epidemic: According to our estimates, they precluded about 84% (499.3K) of the COVID-19 cases and about 66% (5.4K) of the related fatalities that would have occurred without SD policies within three weeks (until April 2). While large effects emerged across the entire population, the relative effects were smaller for the oldest age group.

From a broader perspective, we have made a step towards quantifying the effects of SD policies. At the same time, we believe that we still need a more comprehensive evaluation of such policies. First, the evidence on confirmed cases may not capture the entire impact of the policies on the epidemic spread. One reason is that, although we use high-quality data, not all infections are detected. If the data improve over time, our analysis can be repeated. Second, we estimate the number of confirmed COVID-19 cases and fatalities avoided within three weeks after the policies’ introduction. In the medium or long run, the picture might change in many ways. On the one hand, some of the avoided infections may have only been shifted to a later time. On the other hand, medical capacities may have been exceeded without SD policies, resulting in even higher numbers of fatalities. Third, our analysis identifies the joint effects of all elements of the policy response. Ideally, future studies find ways to disentangle the effects of appeals for voluntary SD, school closures, and contact bans. To shed light on these and other issues, researchers could adapt the event-study approach to new data and settings, including the policies’ removals.

## Supporting information

S1 File (PDF)
