# Supplementary material for: How effective are social distancing policies? Evidence on the fight against COVID-19
Source: PLoS One. 2021 Sep 22;16(9):e0257363. doi: 10.1371/journal.pone.0257363 (PMC8457454; doi:10.1371/journal.pone.0257363)
Supplement: S1 File — (PDF) [file pone.0257363.s001.pdf]

Supporting information: S1 File

Paper: How Effective Are Social Distancing Policies? Evidence on the Fight Against COVID-19

Authors: Ulrich Glogowsky, Emanuel Hansen, and Simeon Schächtele

S.1 Data structure and estimation

Structure of the Dataset: Our analysis uses panel data. The data contains one observation of each outcome variable for each of the 401 German districts i and each day t. Table S1 shows an excerpt of our dataset. Particularly, it displays the cumulative confirmed cases C_{it} in two districts $i = 138$ (Hersfeld-Rotenburg) and $i = 187$ (Hohenlohekreis) at four days in March 2020 (3/5, 3/11, 3/12, and 3/18).

Data Preparation for Estimation: Before we can apply our estimation approach, we process the data in the following way. First, we identify the district-specific outbreak date and record it in a variable od_i (see Table S1). Specifically, we define the outbreak date in district i as the first day when ten cases had occurred within two weeks. As can be seen in Table S1, the outbreak in district 138 occurred on March 12, while the outbreak in district 187 occurred one week earlier, on March 5. Second, we create a new variable et_{it} that measures the *epidemic time* for each observation in district i at time t, defined as the number of days relative to the district-specific outbreak date od_i. Third, we create a set of dummy variables $1[et_{it} = k]$ for each epidemic time k in $\{-41, -40, \ldots, -1, 0, 1, \ldots, 60, 61\}$. The dummy variable $1[et_{it} = k]$ takes the value 1 if the epidemic time of an observation equals k and the value 0 otherwise. Fourth, we create a set of time dummy variables $1[t = j]$ with j in $\{2/15, 2/16, \ldots, 4/18, 4/19\}$. The dummy variable $1[t = j]$ takes the value 1 if the observation date t equals j and 0 otherwise. As the policy change occurred simultaneous in all German districts, this second set of dummies captures the *event time* of an observation (i.e., the date relative to Merkel's appeal). Finally, to capture the potentially exponential nature of the data, we transform the cumulative confirmed cases C_{it} using the *inverse hyperbolic sine* (IHS) to compute our outcome variable Y_{it}. We do the same with our fatalities measure.

Table S1. Data structure and excerpt for illustration

obs	i	t	C_{it}	od_i	et_{it}	$1[et_{it} = 0]$	$1[et_{it} = 6]$	$1[t = 3/11]$	$1[t = 3/12]$	Y_{it}
6596	138	3/5	0	3/12	-7	0	0	0	0	0.0
⋮	⋮	⋮	⋮	⋮	⋮	⋮	⋮	⋮	⋮	⋮
6602	138	3/11	6	3/12	-1	0	0	1	0	2.49
6603	138	3/12	12	3/12	0	1	0	0	1	3.18
⋮	⋮	⋮	⋮	⋮	⋮	⋮	⋮	⋮	⋮	⋮
6609	138	3/18	31	3/12	6	0	1	0	0	4.13
⋮	⋮	⋮	⋮	⋮	⋮	⋮	⋮	⋮	⋮	⋮
8948	187	3/5	14	3/5	0	1	0	0	0	3.33
⋮	⋮	⋮	⋮	⋮	⋮	⋮	⋮	⋮	⋮	⋮
8954	187	3/11	41	3/5	6	0	1	1	0	4.41
8955	187	3/12	51	3/5	7	0	0	0	1	4.63
⋮	⋮	⋮	⋮	⋮	⋮	⋮	⋮	⋮	⋮	⋮
8961	187	3/18	211	3/5	13	0	0	0	0	6.05

Estimation Approach: Equipped with this data, we estimate with ordinary least squares (OLS) the multivariate regression model (1),

$$Y_{it} = \sum_{k \in \{\dots,-1,0,1,\dots\}} \alpha_k \cdot 1[et_{it} = k] + \sum_{j \neq 3/11} \beta_j \cdot 1[t = j] + \varepsilon_{it},$$

where the dependent variable Y_{it} is, for example, given by the IHS-transformed number of infections in district i at date t. By contrast, the explanatory variables are given by the dummies for the epidemic time, $1[et_{it} = k]$, and the event time, $1[t = j]$. We follow the convention to omit the event time dummy for March 11, the day before the policy change took place. The error term is denoted by ε_{it}. Moreover, we cluster the standard errors at the district level.

Intuition of Our Estimation Strategy: To illustrate the role of each set of variables, first, consider a simple thought experiment: Assume that we were in a world without any policy change and would estimate the following simplified regression model:

$$Y_{it} = \sum_{k \in \{\dots,-1,0,1,\dots\}} \alpha_k \cdot 1[et_{it} = k] + \varepsilon_{it}.$$

This model, hence, only includes the first set of epidemic-time dummies. Then, each of the OLS estimates $\hat{\alpha}_k$, $\hat{\alpha}_{k+1}$, ... represents a non-parametric estimate of the non-linear epidemic spread in district i after the district-specific outbreak date od_i. Specifically, the estimate $\hat{\alpha}_k$ would reflect the expected value of the dependent variable in district i at epidemic time $et_{it} = k$, $E[Y_{it} \mid et_{it} = k] = \hat{\alpha}_k$.

Second, assume that there was a policy change at March 12. If we would nevertheless run the reduced model with epidemic time dummies only, we would estimate the average spread of the epidemic across the entire set of districts (i.e., those that face outbreaks before and those that face outbreaks after the policy change). This model, hence, reveals how fast the epidemic spreads on average, but it is silent on the effects of the SD policies.

Third, we want to identify how the SD polices affected the spread of the epidemic. To that end, we estimate the full model (1) that takes into account both the epidemic time dummies and the event time dummies. This model separately estimates the epidemic spread that would have occurred without policy change – captured by the α parameters – and the policy effects – captured by the β parameters. To see this, consider a simple example. The expected value of the outcome in district i with epidemic time k at some date after the policy change – say, on March 17 – is given by $E[Y_{it} \mid t = 3/17, et_{it} = k] = \hat{\alpha}_k + \hat{\beta}_{3/17}$. The predicted outcome in the counterfactual world without policy change is given by $E[Y_{it} \mid t = 3/11, et_{it} = k] = \hat{\alpha}_k$. This equals the expected value of Y_{it} in a district i with the same epidemic time $et_{it} = k$ at March 11, the day before the policy change. Correspondingly, an estimate of the policy effect on March 17 is given by the difference between the expected values (conditional means), $E[Y_{it} \mid t = 3/17, et_{it} = k] - E[Y_{it} \mid t = 3/11, et_{it} = k] = \hat{\beta}_{3/17}$. Figure S13 below provides a graphical illustration of this estimation approach and our model fit.

Note that we can estimate the α parameters and β parameters separately because our data involves variation in the *epidemic time* across observations from each calendar date t and vice versa (see Table S1).

Identifying Assumption: Our estimation relies on the identifying assumption that, without SD policies, the epidemic spread in districts with outbreaks after the intervention would have been similar to the one in districts with outbreaks before the intervention (parallel trends). While we cannot test this assumption directly, the

inclusion of event-time dummies from before the intervention allows for a simple and common verification of its plausibility: For any event time j before March 11, our estimate $\hat{\beta}_j$ should be close to zero. We provide further plausibility checks in Table S2, where we consider potential correlations between outbreaks dates and epidemic spreads.

Appeal of Non-Parametric Approach: Notably, our event study provides a non-parametric estimate of the effects of the German SD policies at each day after their implementation $\hat{\beta}_{3/12}$ to $\hat{\beta}_{4/2}$. Hence, we do not impose exponential growth upfront. We find that the effect of the policies on mobility behavior immediately becomes significant and grows initially, but starts to shrink after the end of March. By contrast, the policies' effects on cases and fatalities only become significant from March 18 on (i.e., six days after Merkel's appeal) and continue to grow over time.

Table S2. Correlations between outbreaks and progression of outcome

**Panel A. Confirmed cases measured in IHS units after:**							
	1 day	2 days	3 days	4 days	5 days	6 days	7 days
Local outbreak date	0.002	-0.001	0.001	0.007	0.017	0.021	0.016
	(0.004)	(0.004)	(0.005)	(0.006)	(0.009)	(0.012)	(0.016)
	[0.633]	[0.830]	[0.903]	[0.272]	[0.051]	[0.081]	[0.315]
**Panel B. Fatalities measured in IHS units after:**							
	1 day	2 days	3 days	4 days	5 days	6 days	7 days
Local outbreak date	-0.005	-0.002	0.001	0.013	0.006	-0.003	0.005
	(0.007)	(0.008)	(0.009)	(0.011)	(0.015)	(0.017)	(0.022)
	[0.508]	[0.814]	[0.896]	[0.239]	[0.675]	[0.860]	[0.826]
N	337	321	294	265	216	178	138

Notes: This table tests if the epidemic spread depends on the date of the local outbreak. The logic is simple: If, for example, districts with earlier outbreaks experienced a more rapid spread of COVID-19, then it is unlikely that these districts constitute a valid counterfactual. The details of the tests are as follows: First, we restrict our sample to observations before the implementation of SD policies. Second, we estimate bivariate OLS regressions of local cumulative COVID-19 cases (Panel A) and deaths (Panel B) X days after the local outbreak on the date of the local outbreak and a constant. Each column displays a different $X \in (1, 2, 3, 4, 5, 6, 7)$. As in the main model, the dependent variables are the inverse hyperbolic sine (IHS) transformation of the local cumulative case or death count. The table presents heteroscedasticity-robust standard errors in parentheses and p-values in brackets. With a Bonferroni correction for multiple testing, no correlation is significant (even at the ten percent level). To see this, note that all p-values exceed the threshold 0.10/7=0.0143 (multiple testing within a panel) and a fortiori also exceed 0.10/14=0.0071 (multiple testing across panels).

Fig S1. Epidemic spread and distancing policies in Germany (April 19)

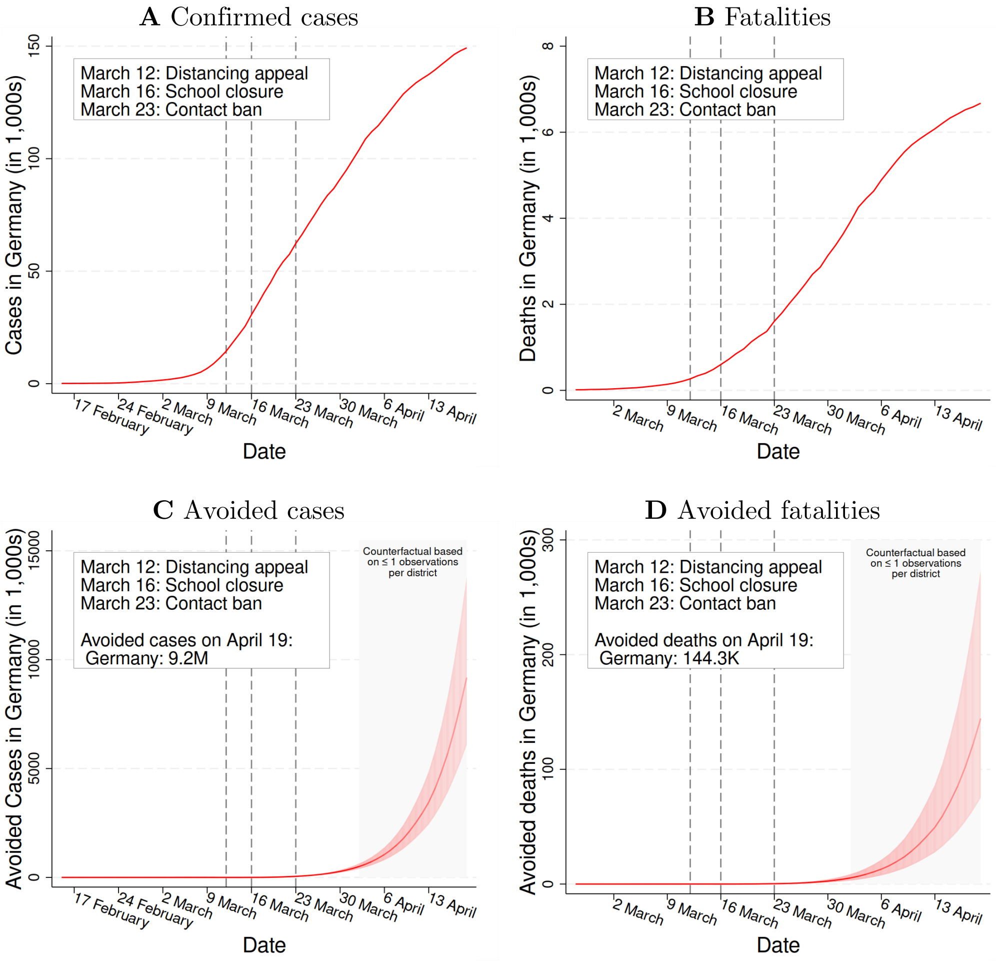

Notes: This figure depicts the epidemic spread and the effect of social distancing policies in Germany until April 19. It shows cumulative counts of confirmed COVID-19 cases (Subfigure S1A) and fatalities (Subfigure S1B) before and after the introduction of social distancing policies. On top of that, Subfigure S1C depicts our estimates on the number of cases avoided by social distancing policies. By contrast, Subfigure S1D focuses on avoided fatalities. The three vertical lines mark the Chancellor's appeal for social distancing (March 12), the nationwide school closures (March 16), and the nationwide contact bans (March 23). The dashed lines represent 95% confidence intervals based on district-level-clustered standard errors.

Fig S2. Social distancing effects in the extended period

A Social distancing effect on conf. cases **B** Social distancing effect on fatalities

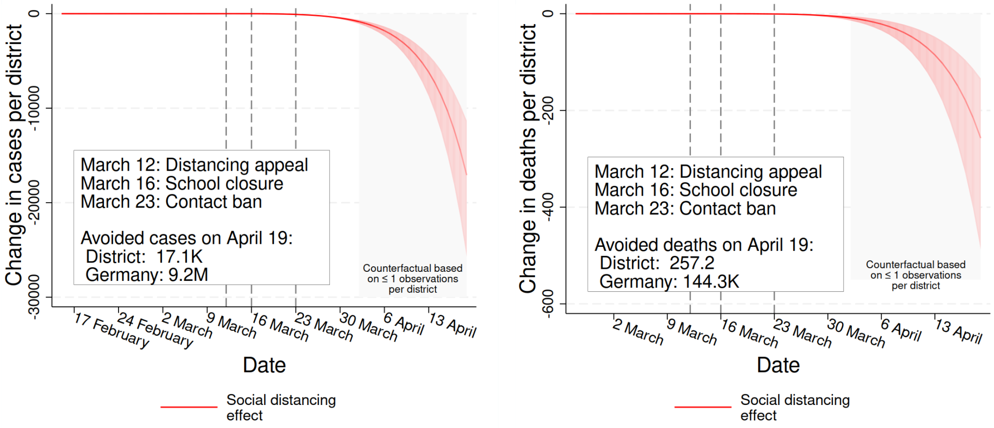

Notes: This figure shows social distancing effects until April 19. Subfigure S2A focuses on confirmed cases and Subfigure S2B on fatalities. The three vertical lines mark the Chancellor's appeal for social distancing (March 12), the nationwide school closures (March 16), and the nationwide contact bans (March 23). The dashed lines represent 95% confidence intervals based on district-level-clustered standard errors.

Fig S3. Heterogeneity of the social distancing effects on fatalities (April 2)

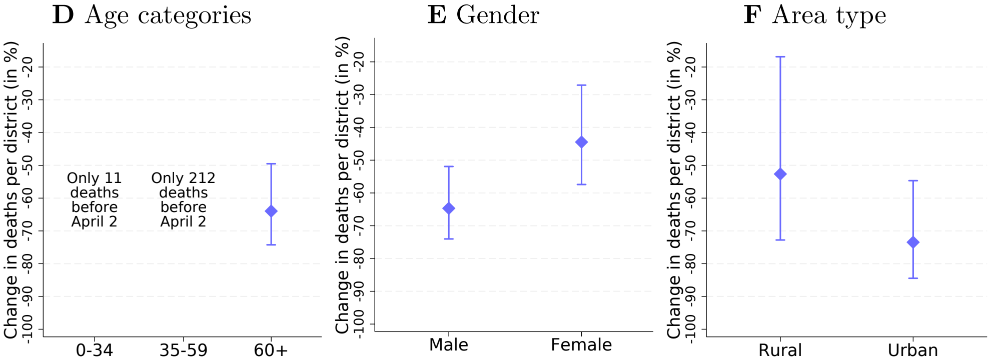

Notes: This figure shows how the effect of the nationwide social distancing policies on April 2 differed (a) across age groups, (b) by gender, and (c) between urban and rural districts. The estimates rely on sample splits and show the percentage of fatalities avoided by the nationwide policies. The vertical lines represent 95% confidence intervals based on district-level-clustered standard errors.

Fig S4. Heterogeneity of the social distancing effects (April 19)

Confirmed cases

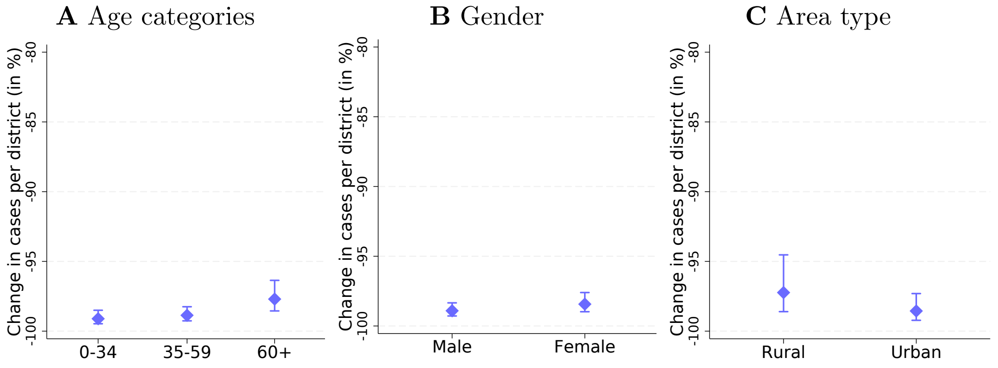

Fatalities

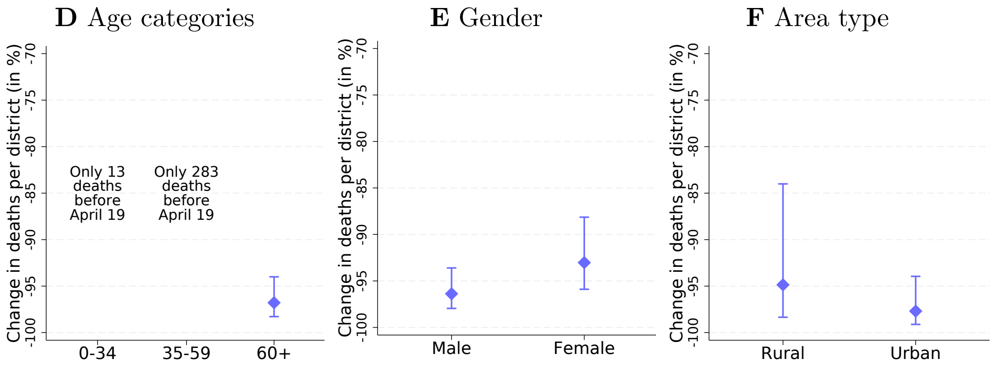

Notes: This figure shows how the effect of the nationwide social distancing policies on April 19 differed (a) across age groups, (b) by gender, and (c) between urban and rural districts. The estimates rely on sample splits and show the percentage of cases (first row) and fatalities (second row) avoided by the nationwide policies. The vertical lines represent 95% confidence intervals based on district-level-clustered standard errors.

Fig S5. Confirmed COVID-19 cases in Germany by groups

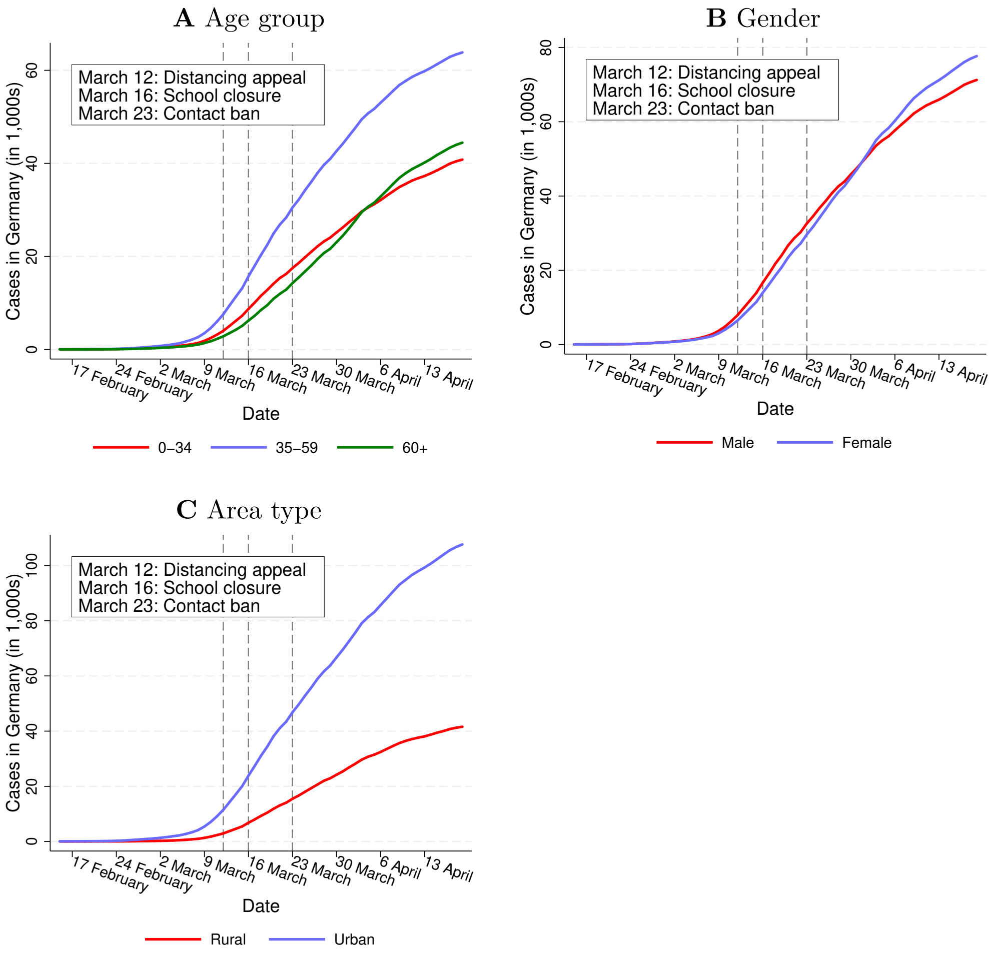

Notes: This figure shows cumulative counts of confirmed COVID-19 cases before and after the introduction of social distancing policies. It decomposes COVID-19 cases (a) across age groups, (b) by gender, and (c) between urban und rural districts.

Fig S6. COVID-19 fatalities in Germany by groups

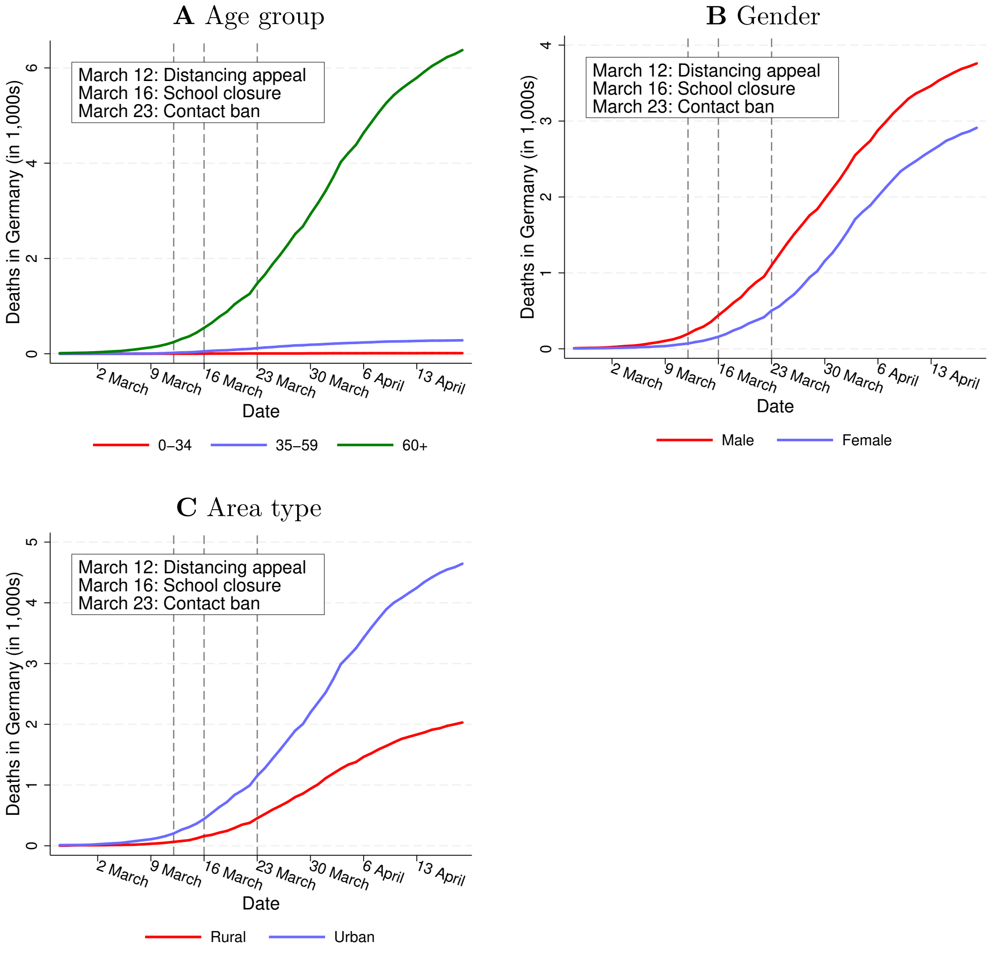

Notes: This figure shows cumulative counts of COVID-19 fatalities before and after the introduction of social distancing policies. It decomposes fatalities (a) across age groups, (b) by gender, and (c) between urban und rural districts.

Fig S7. Distribution of district-specific outbreaks

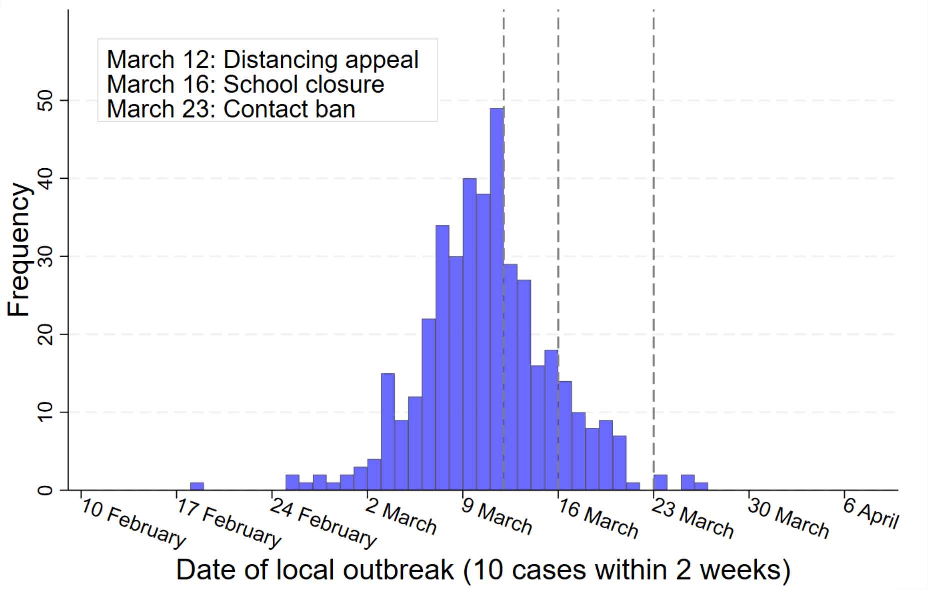

Notes: This figure shows how the district-specific COVID-19 outbreaks are distributed over time. Local outbreak dates are defined as the first day when ten confirmed COVID-19 cases have occurred within the last two weeks (where occurred refers to the date of the first symptoms or, if not available, the registry date of the case).

Fig S8. Social distancing effects under diff. outbreak definitions (April 2)

A Reduction in mobility

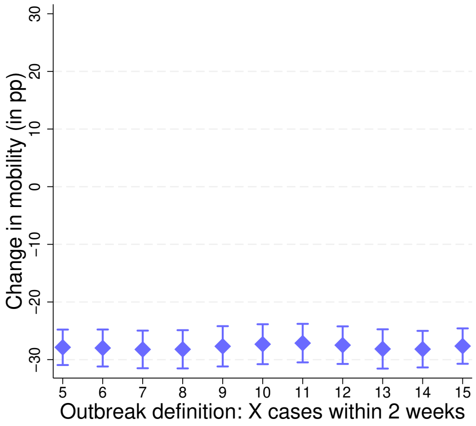

B Avoided cases in Germany

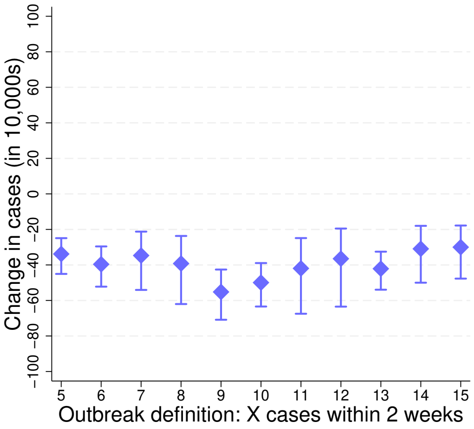

C Avoided fatalities in Germany

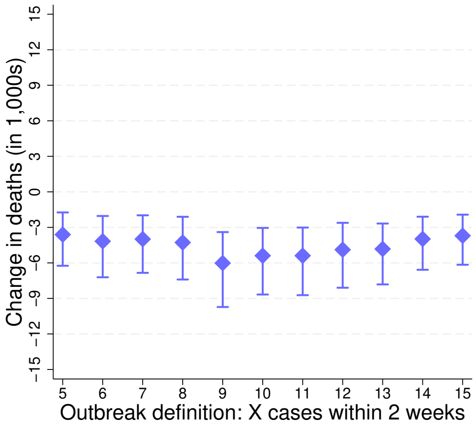

D Baseline definition of local outbreaks

Our baseline definition of an outbreak is the occurrence of ten cases in two weeks. By considering two weeks (instead of a shorter period), we ensure that we account for outbreaks that take some days to progress. Recap that COVID-19 has an incubation period of 2-14 days. Furthermore, we use the ten-cases criterium because the probability that a district registers a new case sharply increases for low case numbers within two weeks and approaches 100% after the tenth case.

Notes: This figure shows social distancing effects on April 2 for different local outbreak definitions. Particularly, it varies how many district-specific COVID-19 cases within two weeks define an outbreak. The figure presents results for different outcome variables: Subfigure S8A focuses on mobility behavior, Subfigure S8B on the total number of avoided cases, and Subfigure S8C on the total number of avoided fatalities. Subfigure S8D discusses our baseline outbreak definition. The vertical lines represent 95% confidence intervals based on district-level-clustered standard errors.

Fig S9. Social distancing effects under diff. outbreak definitions (April 19)

A Reduction in mobility

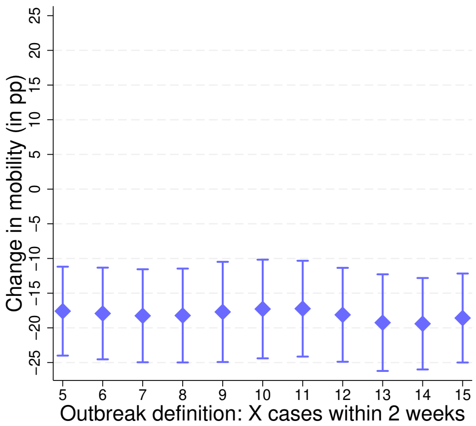

B Avoided cases in Germany

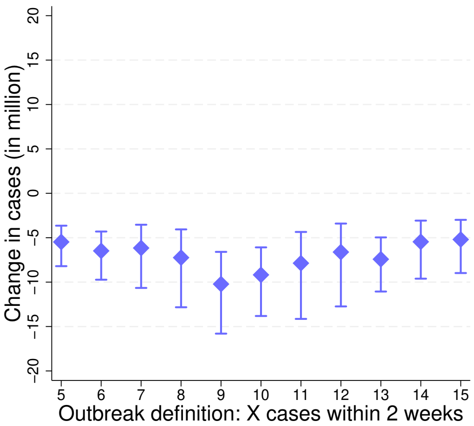

C Avoided fatalities in Germany

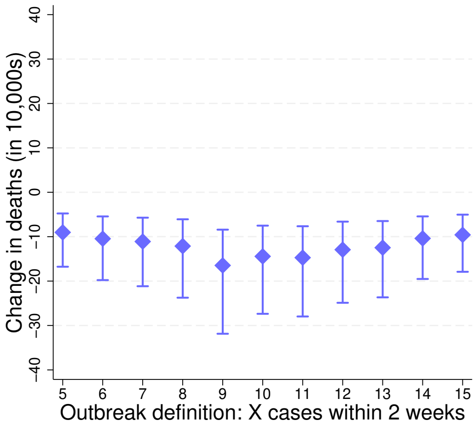

D Baseline definition of local outbreaks

Our baseline definition of an outbreak is the occurrence of ten cases in two weeks. By considering two weeks (instead of a shorter period), we ensure that we account for outbreaks that take some days to progress. Recap that COVID-19 has an incubation period of 2-14 days. Furthermore, we use the ten-cases criterium because the probability that a district registers a new case sharply increases for low case numbers within two weeks and approaches 100% after the tenth case.

Notes: This figure shows social distancing effects on April 2 for different local outbreak definitions. Particularly, it varies how many district-specific COVID-19 cases within two weeks define an outbreak. Further, the figure presents results for different outcome variables: Subfigure S9A focuses on mobility behavior, Subfigure S9B on the total number of avoided cases, and Subfigure S9C on the total number of avoided fatalities. Subfigure S9D discusses our baseline outbreak definitions. The vertical lines represent 95% confidence intervals based on district-level-clustered standard errors.

Fig S10. Further robustness tests for the social distancing effect (April 2)

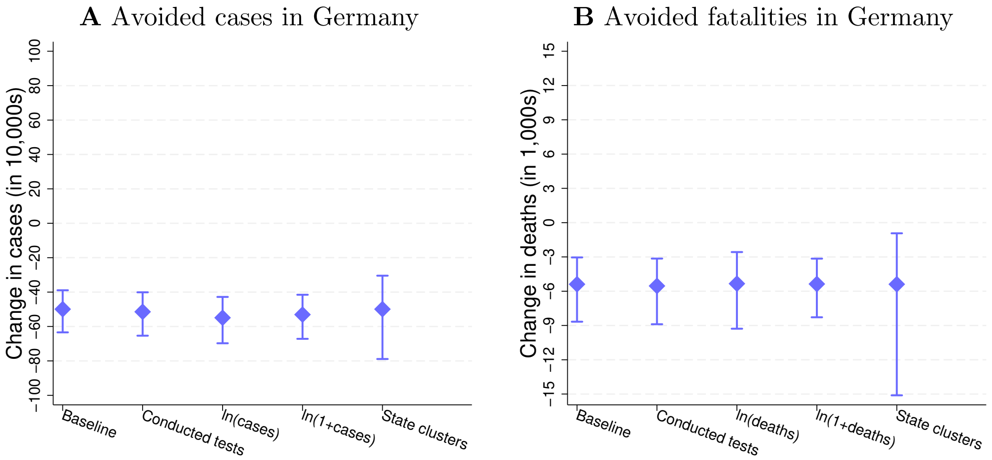

Notes: This figure presents the results of several robustness checks. Subfigure S10A focuses on avoided cases (April 2). Subfigure S10B presents the results for avoided fatalities (April 2). Both subfigures consider five specifications: First, for comparison, they show the baseline specification presented in the main text. The second specification controls for the number of conducted COVID-19 tests per capita. In the third specification, we drop observations with zero counts and take logs of the outcome. The fourth specification keeps observations with zero counts and applies the commonly used $\ln(1+y)$ outcome transformation. The specifications one to four cluster standard errors on the district level. By contrast, specification five shows state-level-clustered standard errors for the baseline estimate. The vertical lines represent 95% confidence intervals.

Fig S11. Further robustness tests for the social distancing effect (April 19)

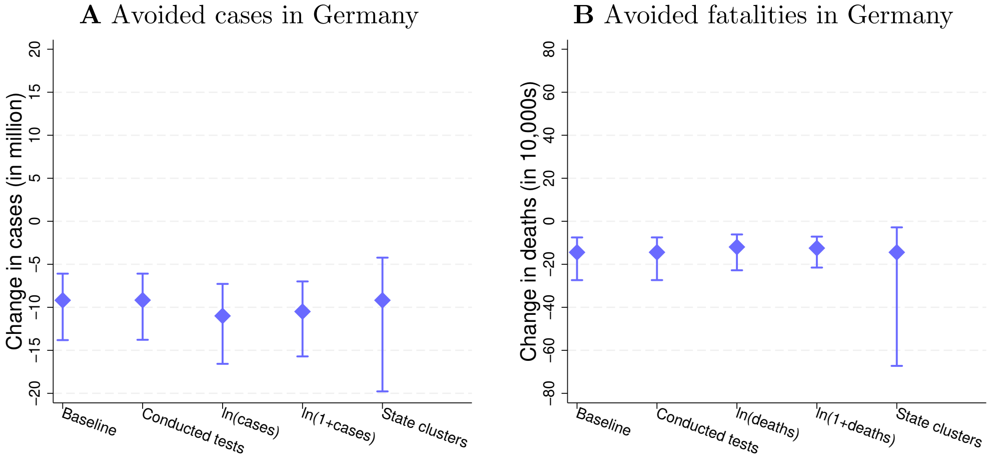

Notes: This figure presents the results of several robustness checks. Subfigure S11A focuses on avoided cases (April 19). Subfigure S11B presents the results for avoided fatalities (April 19). Both subfigures consider five specifications: First, for comparison, they show the baseline specification presented in the main text. The second specification controls for the number of conducted COVID-19 tests per capita. In the third specification, we drop observations with zero counts and take logs of the outcome. The fourth specification keeps observations with zero counts and applies the commonly used $\ln(1 + y)$ outcome transformation. The specifications one to four cluster standard errors on the district level. By contrast, specification five shows state-level-clustered standard errors for the baseline estimate. The vertical lines represent 95% confidence intervals.

Fig S12. The social distancing effect on the reproduction number

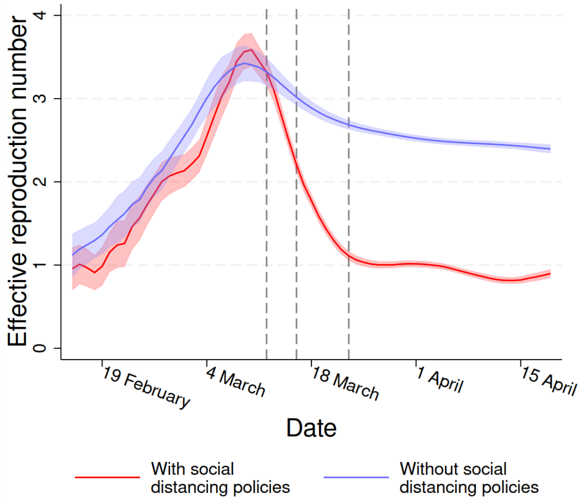

Notes: This figure shows how social distancing policies affect the effective reproduction number. To generate this figure, we estimate a version of model (2) that uses the smoothed 7-day district-specific effective reproduction number as an outcome. To calculate the reproduction number, we follow the official methodology of the Robert Koch Institute [33]. The dashed lines represent 95% confidence intervals based on district-level-clustered standard errors.

Fig S13. Estimating the world without social distancing from the raw data

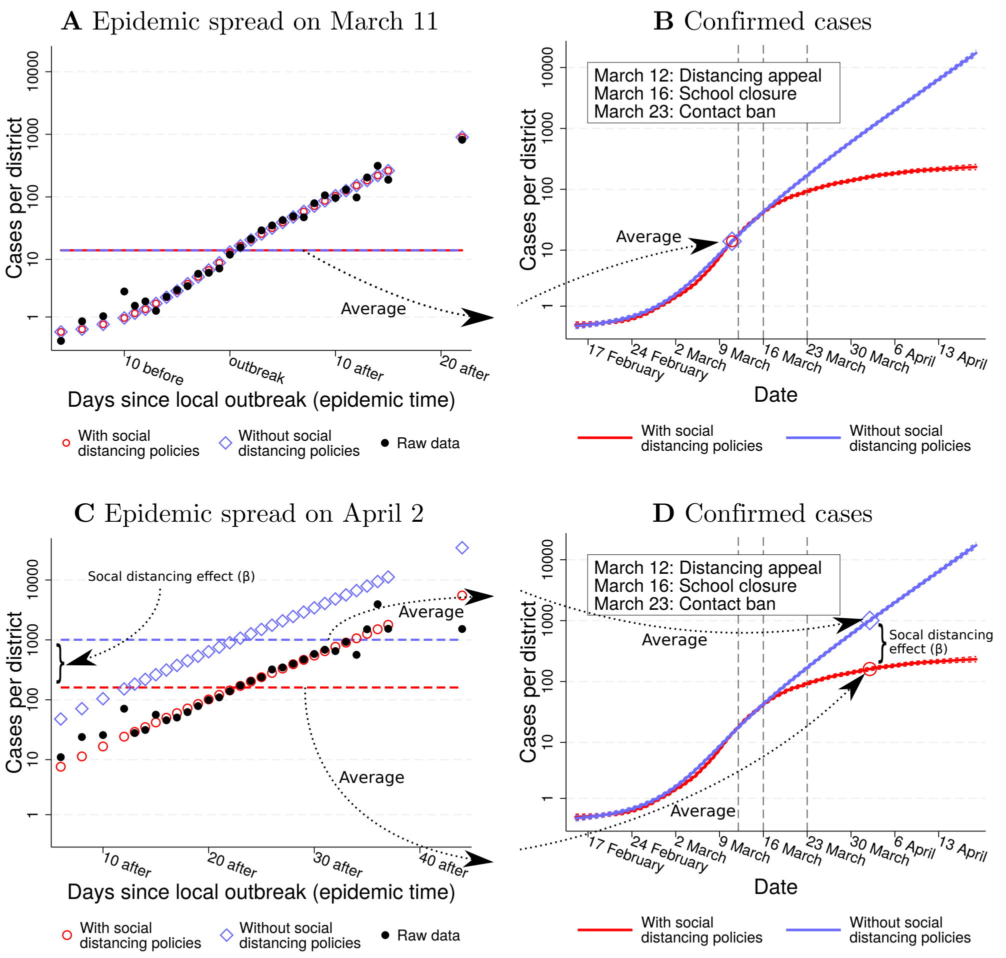

Notes: This figure illustrates our estimation approach graphically. The top (bottom) panel shows how confirmed cases on March 11, 2020 (April 2), enter the estimated evolution of cases. All subfigures represent cases per district (IHS means) on a logarithmic scale. Subfigures A and C show cases conditional on epidemic time. Black circles indicate raw data. Blue diamonds represent the epidemic time dummies estimated from (1) and constitute the no-SD policies prediction. Red circles indicate predicted cases with SD policies. For the omitted base day March 11, blue and red shapes coincide by construction. For April 2, the counterfactual values (blue diamonds) differ from the SD values (red circles) due to the SD effect: It corresponds to the mean deviation of April 2 cases from the cases of districts with the same epidemic times before SD policies. Subfigure A (C) suggests that the model fits the data on March 11 (April 2) very well. Despite the model not imposing constant growth, the predicted values almost lie on an straight line. The averages of the predictions in Panel A (C) (i.e., the horizontal dashed lines) form the per district values on March 11 (April 2) represented in Subfigure B (D).

Fig S14. Apple mobility trends for Germany

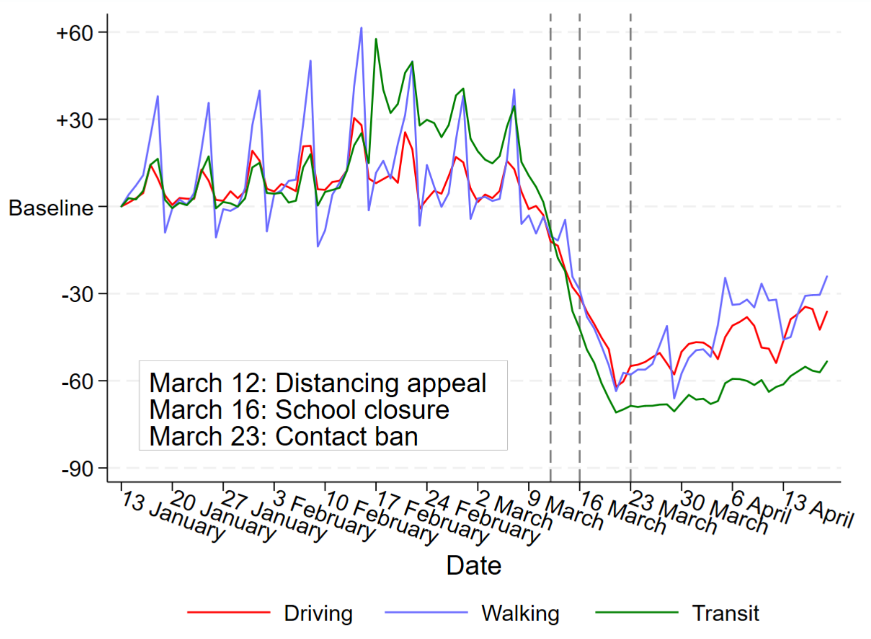

Notes: This figure shows percentage changes in the number of Apple Maps requests for several German cities and districts. The values are measured relative to a baseline date (Jan 13, 2020). The three vertical lines mark the Chancellor's appeal for social distancing (March 12), the nationwide school closures (March 16), and the nationwide contact bans (March 23). Data: `https://www.apple.com/covid19/mobility`

Fig S15. Google mobility trends for Germany

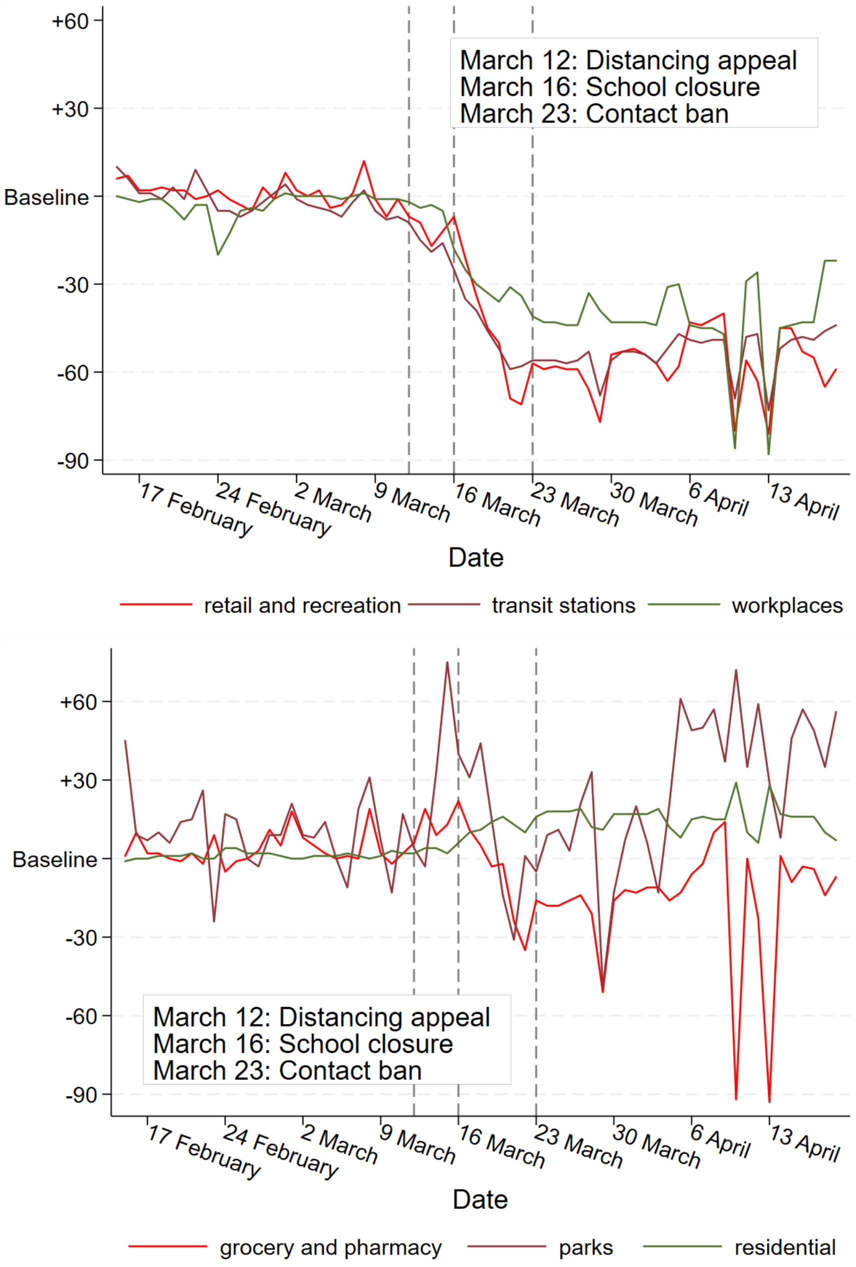

Notes: This figure shows percentage changes in a Google metric based on visits and length of stay at different places in Germany. The baseline is the median value, for the corresponding day of the week, during the 5-week period Jan 3-Feb 6, 2020. The upper figure presents location categories with large reductions in mobility. The bottom figure shows, with more volatility, increases for some places. The downward spikes on April 10 and 13 are due to Easter holidays in Germany. Data: `https://www.google.com/covid19/mobility/`.

Fig S16. Google-search trends for COVID-19 and SD policies

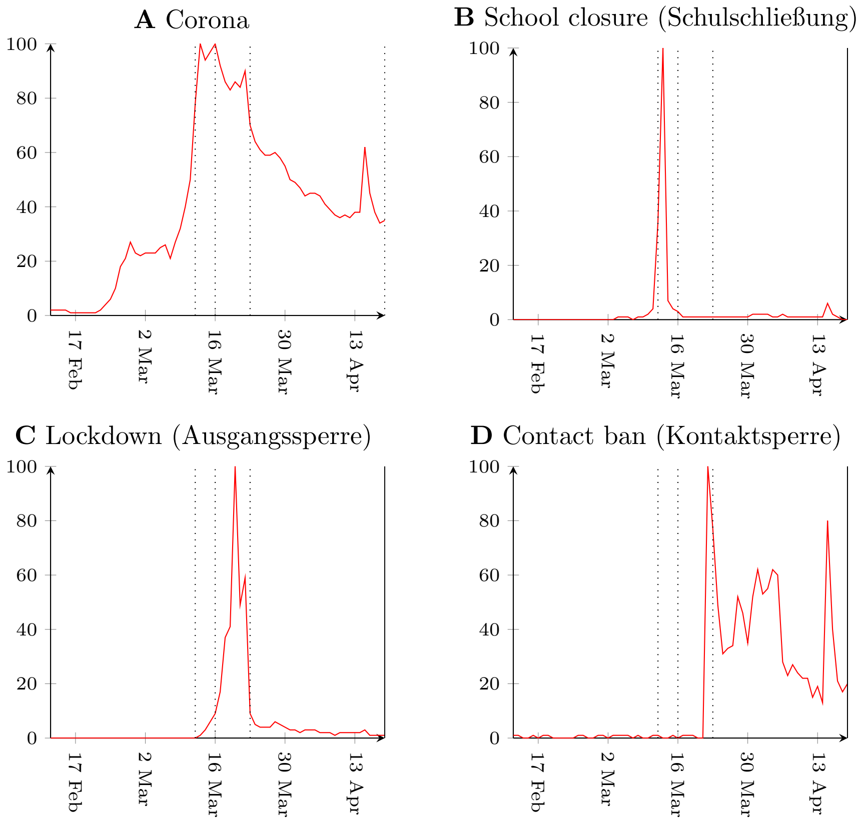

Notes: This figure shows how the relative frequency of Google searches for keywords related to COVID-19 and the German SD policies evolved from February 12 to April 19 in Germany. For each keyword, the maximum frequency of searches on a day is normalized to 100, and the frequencies on all other days are measured relative to this maximum. The three vertical lines mark the Chancellor's appeal for social distancing (March 12), the nationwide school closures (March 16), and the nationwide contact bans (March 23). Data: `https://trends.google.de/`.

Fig S17. Google-search trends for mobility-related keywords

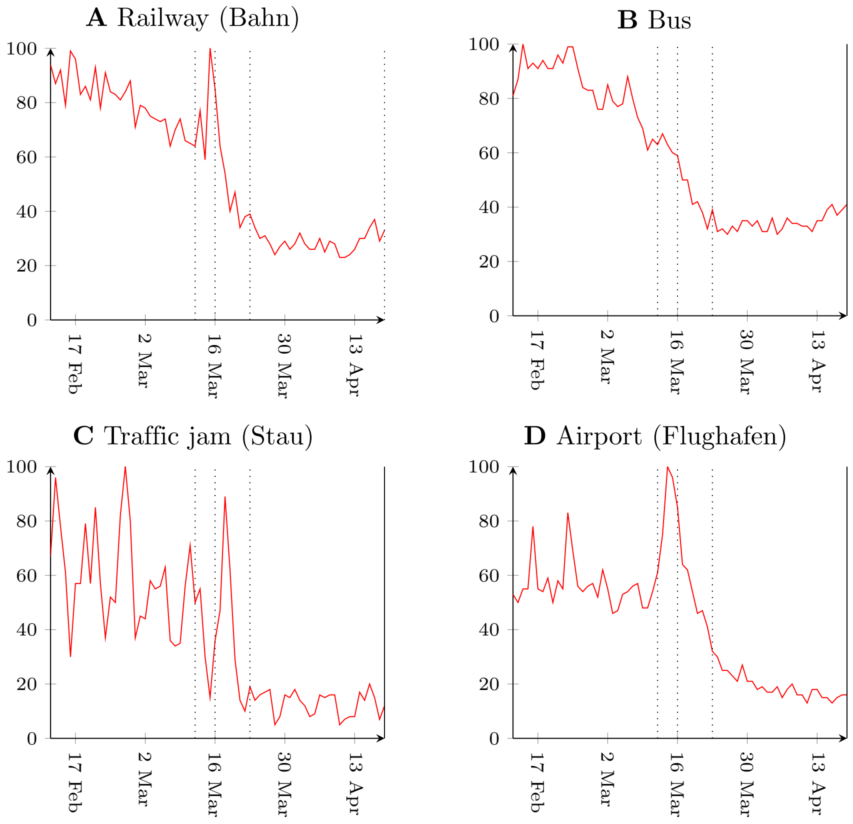

Notes: This figure shows how the relative frequency of Google searches for mobility-related keywords evolved from February 12 to April 19 in Germany. For each keyword, the maximum frequency of searches on a day is normalized to 100, and the frequencies on all other days are measured relative to this maximum. The three vertical lines mark the Chancellor's appeal for social distancing (March 12), the nationwide school closures (March 16), and the nationwide contact bans (March 23). Data: `https://trends.google.de/`.

Fig S18. Google-search trends for work- and catering-related keywords

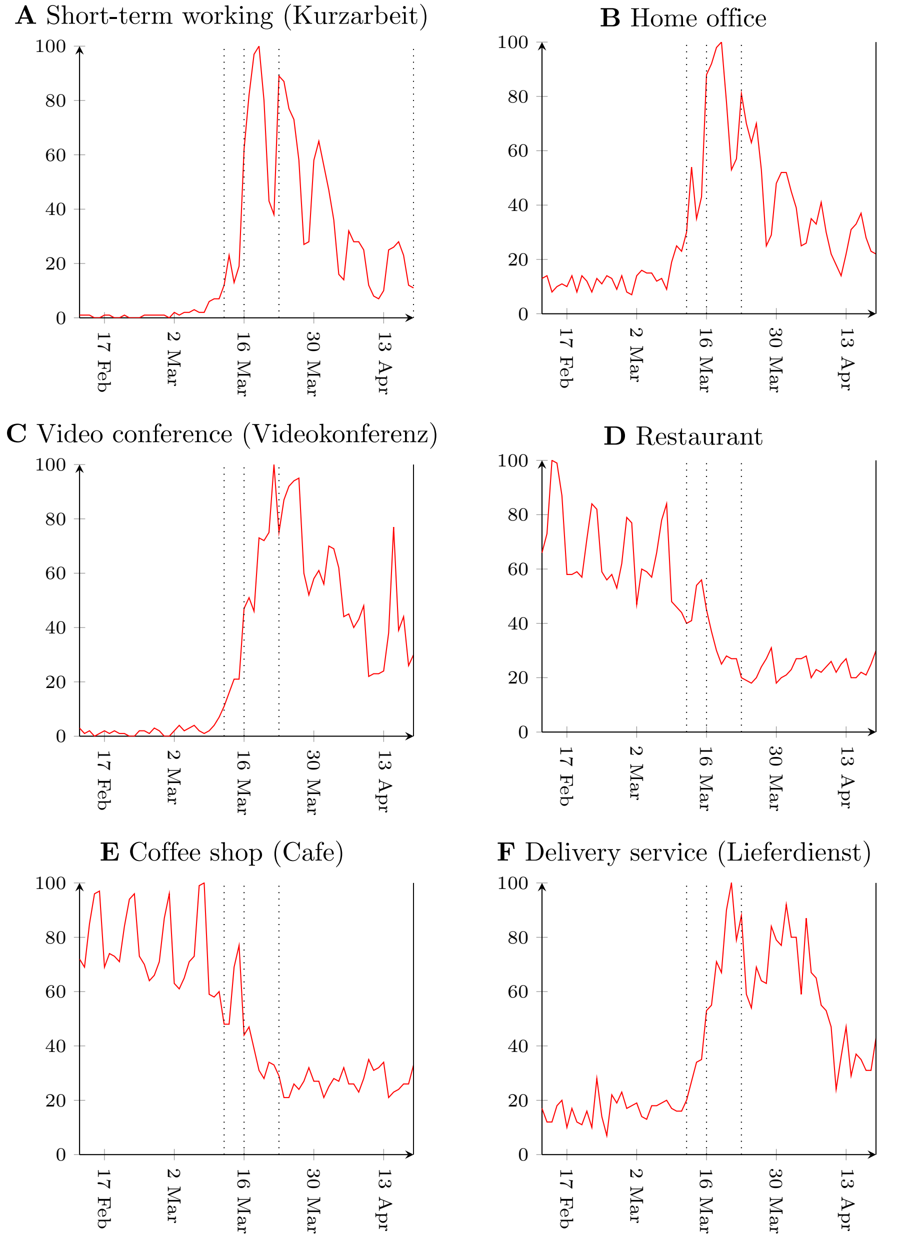

Notes: This figure shows how the relative frequency of Google searches for work-related and catering-related keywords evolved from February 12 to April 19 in Germany. For each keyword, the maximum frequency of searches on a day is normalized to 100, and the frequencies on all other days are measured relative to this maximum. The three vertical lines mark the Chancellor's appeal for social distancing (March 12), the nationwide school closures (March 16), and the nationwide contact bans (March 23). Data: `https://trends.google.de/`.

Fig S19. Google-search trends for leisure time activities

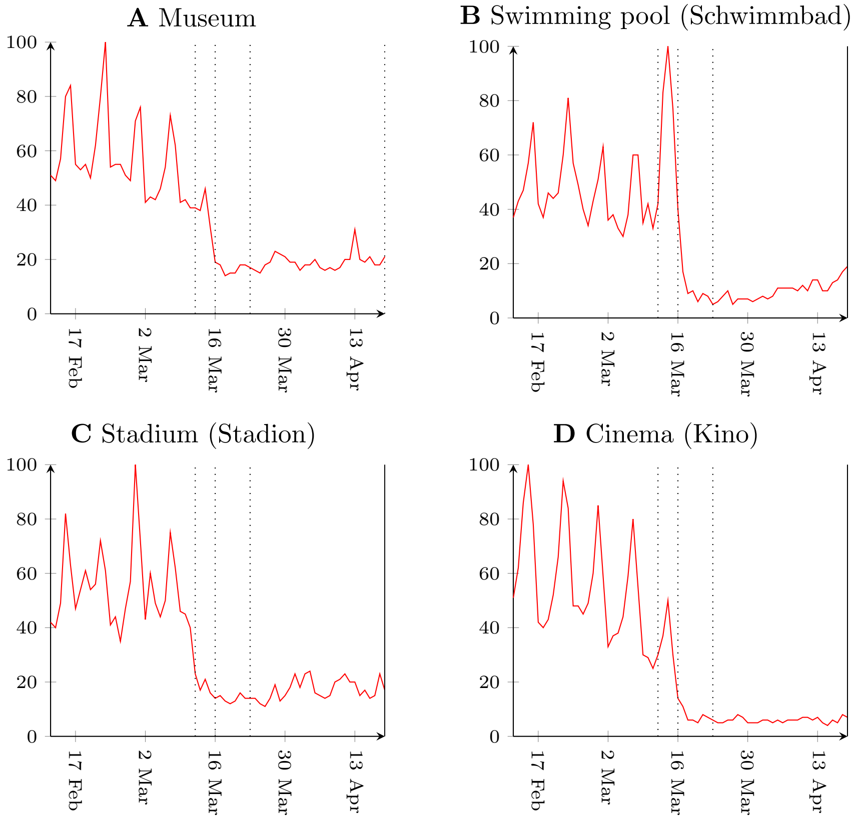

Notes: This figure shows how the relative frequency of Google searches for keywords related to leisure time activities evolved from February 12 to April 19 in Germany. For each keyword, the maximum frequency of searches on a day is normalized to 100, and the frequencies on all other days are measured relative to this maximum. The three vertical lines mark the Chancellor's appeal for social distancing (March 12), the nationwide school closures (March 16), and the nationwide contact bans (March 23). Data: `https://trends.google.de/`.
